# Assessing the Impact of Alopecia on Quality of Life, Depression, and Self-Esteem in Saudi Arabia

**DOI:** 10.7759/cureus.49864

**Published:** 2023-12-03

**Authors:** Nasser M Alzoabi, Hassan M Alsharif, Ahmed M Alawami, Hazim H Habarah, Hussain A Alhawaj, Nouf Bin Rubaian, Jamal M Alqahtani

**Affiliations:** 1 Department of Dermatology, King Fahad University Hospital, Al Khobar, SAU; 2 College of Medicine, Imam Abdulrahman Bin Faisal University, Dammam, SAU; 3 Department of Dermatology, King Fahad University Hospital, Dammam, SAU

**Keywords:** depression, quality of life, saudi arabia, eastern province, self-esteem, hair loss, alopecia

## Abstract

Objectives

The objective of this study is to assess the effects of alopecia on quality of life, depression, and self-esteem in the Eastern Province of Saudi Arabia.

Methods

We made a questionnaire that included sociodemographic data, type of alopecia, medical aid sought, and whether they benefited from it or not. The study uses the Dermatology Life Quality Index, Patient Health Questionnaire-9, and a single-item self-esteem scale to assess the quality of life, depression, and self-esteem, respectively.

Results

The questionnaire was completed by 403 individuals in total, and after applying the exclusion criteria (only Saudis, from the Eastern Province), 231 participants were included in this study. Of the participants, 49.4% had hereditary baldness and only 9.5% benefited from treatment. Of the patients, 52.4% had higher levels of depression, and 18.2% had an effect on their life. Patients with a moderate or greater effect on their lives had a significant relationship with hereditary baldness. Younger age, being female, being married, having lower income, and having hereditary baldness were significantly associated with higher depression levels (p = <0.05). The study found that as age increased, depression levels decreased and self-esteem scores increased. Depression was linked to lower quality of life, while self-esteem was linked to both lower quality of life and higher depression levels. These factors are interrelated, with age influencing their relationship.

Conclusions

The results of the study highlight the significant occurrence of depression and decreased quality of life among patients who have alopecia, particularly those with hereditary baldness. It is crucial to provide psychological assistance and counseling to enhance their mental health and overall wellness.

## Introduction

Alopecia, also known as hair loss, is a condition that has an influence on individuals' quality of life, mental health, and self-esteem. It is not limited to any gender or age group, as both men and women across age ranges can experience it. Multiple factors contribute to the development of alopecia, including genetics, hormonal fluctuations, autoimmune conditions, and certain medical therapies, like chemotherapy [1,2]. Furthermore, alopecia has the potential to cause challenges and can have a detrimental effect on a person’s self-assurance, self-esteem, and perception of themselves [3]. Moreover, it can lead to increased self-awareness and social anxiety, as individuals might feel ashamed or uneasy about their appearance [4]. In Saudi Arabia, the impact of alopecia on people’s quality of life, mental well-being, and self-esteem holds importance due to the social and psychological associations associated with hair loss.

In society, hair has significant cultural importance representing beauty, youth, and vitality. Consequently, hair loss can be viewed as a decline in attractiveness and femininity [3]. In postmenopausal women, the prevalence of androgenetic alopecia rises dramatically. In white Caucasians, women aged 20 to 29 years and 70 to 89 years had a 3% and 29% prevalence, respectively [5,6]. This cultural emphasis on hair can amplify distress and lead to negative self-perception among those facing hair loss.

It is crucial to acknowledge that alopecia transcends geographical boundaries and affects individuals from diverse ethnicities and backgrounds worldwide. For example, in the United States, men aged 18-29 years and 40-49 years have a prevalence of 16% and 53%, respectively. Also in Korea, among men aged 20 to 29 years, the prevalence is 14% [7].

Recognizing the significance of alopecia on individuals is important for many reasons. To begin with, it enables healthcare practitioners to offer assistance and interventions to those who are facing hair loss. These interventions may encompass counseling, participation in support groups, as well as access to treatments like hair restoration therapies or wigs [4].

Moreover, comprehending the ramifications of alopecia can aid in dismantling stigmas, fostering acceptance, and enhancing understanding within society. This, in turn, contributes toward building a supportive community for individuals suffering from alopecia, which is no small number, according to the findings of one study conducted by Alomaish et al. in Jazan, which aimed to estimate the prevalence of alopecia among primary healthcare attendees. It was discovered that out of 729 patients, 483 of them reported having the problem, accounting for 66.3% of the total sample [8].

Neglected or poorly managed alopecia can have implications for individuals’ overall well-being. It can lead to seclusion, as individuals might refrain from engaging in activities and maintaining relationships due to feelings of embarrassment or self-consciousness [9]. Furthermore, it can affect many aspects of one’s life, including personal relationships, professional performance, and overall life satisfaction. Moreover, research has indicated that individuals with alopecia are more prone to experiencing symptoms of depression and anxiety. Hence, it is imperative to address the repercussions of alopecia and offer appropriate support and interventions that enhance an individual’s well-being and overall life quality [3].

To conclude, alopecia can have an effect on the quality of life, mental health, and self-esteem of an individual. This impact holds significance in Saudi Arabia, given the social and psychological implications surrounding hair loss. Recognizing the consequences of alopecia plays a role in offering pertinent support and interventions to those grappling with alopecia with the ultimate aim of fostering an inclusive and compassionate society. When left untreated or poorly managed, alopecia can result in seclusion, diminished self-assurance, and compromised welfare. Hence, it becomes imperative to address the ramifications of alopecia, by providing support and interventions that enhance the quality of life and overall well-being of affected individuals.

This study aimed to assess the effect of alopecia on quality of life, depression, and self-esteem in the Eastern Province of the Kingdom of Saudi Arabia.

## Materials and methods

Study design

The chosen study design for this study is a cross-sectional study, which was chosen to enable us to manage the multiple variables, such as age, sex, marital status, monthly income, educational level, occupation, type of alopecia, whether they sought medical treatment, and if it was beneficial, as well as collect the data required for us to assess the effects of alopecia on health-related quality of life, depression, and self-esteem in a suitable, time-efficient manner.

Population demographics

The study was conducted in the Eastern Province of the Kingdom of Saudi Arabia with the targeted population being Saudis with alopecia of both genders and of various ages.

Sampling technique/size

To conduct this study, voluntary response sampling was used to collect data through an online-based questionnaire. Due to the lack of prevalence studies of alopecia in the Eastern province, we could not determine the population. Therefore, we calculated the sample size using the following formula: we calculated the sample size using a confidence interval of 95%, a margin of error of 5%, a z-score of 1.96, and a standard deviation of 0.5.

Data collection methods

The questionnaire will be available in both the English and Arabic languages. It will ask about the participants sociodemographic data in section 2. In section 3, information about the type of alopecia, as well as medical aid sought and medication use will be obtained. In section 4, quality of life will be assessed using the Dermatology Life Quality Index (DLQI). In section 5, depression will be assessed through the use of Patient Health Questionnaire-9 (PHQ-9). In section 6, the single-item self-esteem scale will be used to assess the self-esteem of the participants.

Instruments

The DLQI is a self-administered 10-question index used to measure how dermatological conditions affect life quality [10]. The PHQ-9 is a self-administered nine-question questionnaire used to assess the degree and severity of depression [11]. The single-item self-esteem scale is a self-administered one-item measure used to assess self-esteem This scale, although made as a shorter alternative to the Rosenberg Self-Esteem Scale, is very reliable and valid as it has very similar predictive similarities [12].

Data analyses

Data were analyzed statistically using SPSS version 26 (IBM Corp., Armonk, NY). To test the relationship between variables, qualitative data was expressed as numbers and percentages, and the chi-squared test (χ2) was used. Quantitative data were expressed as mean and standard deviation (mean ± SD), and non-parametric variables were tested using the Mann-Whitney test. Correlation analysis was performed using Spearman's test, and a p-value of less than 0.05 was considered statistically significant.

## Results

Table 1 shows that the mean age of the studied participants was 38.09 ± 14.62 years, 54.5% were males, 64.5% were married, and 37.2% were employed. Of them, 34.6% had a monthly income <2000 Saudi riyal (SR) and 48.9% had a bachelor's degree. The most common cause of alopecia was hereditary baldness, and 49.4% sought treatment but only 9.5% benefited from it.

**Table 1:** Distribution of studied participants according to their demographic data, causes of alopecia, and seeking treatment and its effect (n = 231)

Variable	No. (%)
Age (years)	38.09 ± 14.62
Gender	
Female	105 (45.5)
Male	126 (54.5)
Marital status	
Widow	1 (0.4)
Single	76 (32.9)
Married	149 (64.5)
Divorced	5 (2.2)
Employment	
Housewife	29 (12.6)
Student	55 (23.8)
Unemployed	17 (7.4)
Retired	44 (19)
Government employee	40 (17.3)
Private sector employee	46 (19.9)
Monthly income (Saudi Riyal)	
<2000	80 (34.6)
2000-<5000	23 (10)
5000-<10000	38 (16.5)
10000-<15000	25 (10.8)
>15000	65 (28.1)
Education	
Primary	1 (0.4)
Middle	7 (3)
Secondary	63 (27.3)
Diploma	27 (11.7)
Bachelor	113 (48.9)
Postgraduate	20 (8.7)
Causes of alopecia	
Malnutrition	44 (19)
Hereditary baldness	119 (51.5)
Telogen effluvium	17 (7.4)
I don't know the type	49 (21.2)
Psoriasis	2 (0.9)
Have you searched for a treatment for this problem?	
No	117 (50.6)
Yes	114 (49.4)
If yes, did you benefit from the treatment?	
No	92 (39.8)
Yes	22 (9.5)

**Figure 1:**
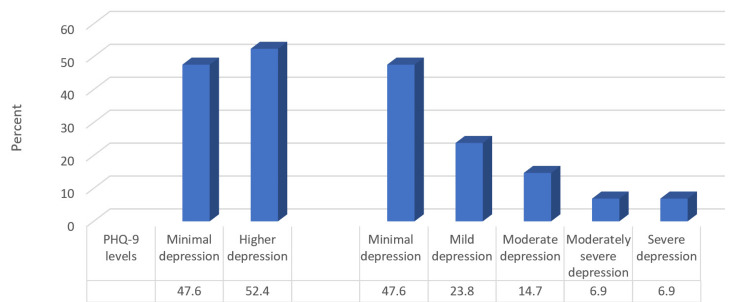
Percentage distribution of studied participants according to depression prevalence and its levels based on the PHQ-9 scores classification (n = 231) PHQ-9: Patient Health Questionnaire-9.

Based on the PHQ-9 scores classification, 47.6% of the participants had minimal depression, and 52.4% had higher levels of depression. While 23.8%, 14.7%, and 6.9% had mild, moderate, and moderately severe or severe depression, respectively (Figure 1).

**Figure 2:**
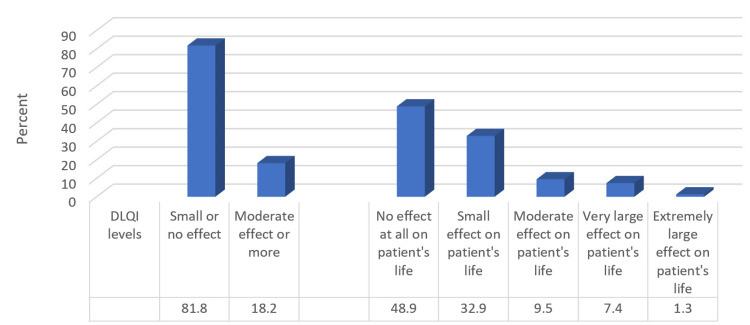
Percentage distribution of studied participants according to dermatology life quality based on the DLQI scores classification (n = 231) DLQI: Dermatology Life Quality Index.

Figure 2 shows that based on the DLQI scores classification, 81.8% had small or no effect on their life, while 18.2% had an effect. In detail, 48.9% had no effect on the patient's life, 32.9% had a small effect, 9.5% had a moderate effect, 7.4% had a very large effect, and 1.3% had an extremely large effect on the patient's life.

**Figure 3:**
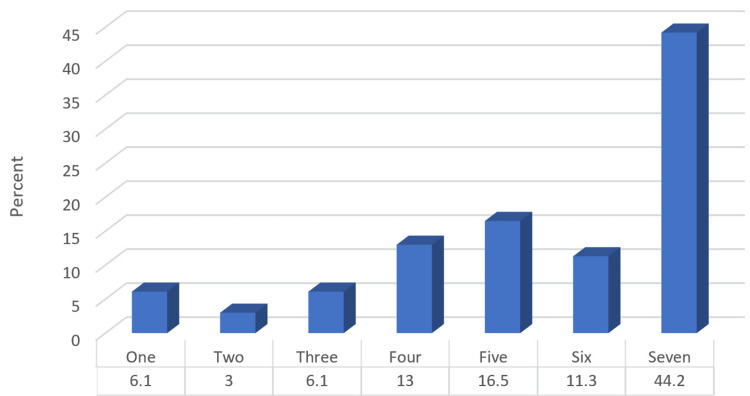
Percentage distribution of studied participants according to their self-esteem

The mean self-esteem score was 5.41 ± 1.82, where most of the participants (44.2%) rated their self-esteem with a score of 7 (Figure 3).

Table 2 shows that patients who had a moderate effect or more on their lives had a significantly higher percentage of those having hereditary baldness (p < 0.05). On the other hand, a non-significant relationship was found between DLQI levels and participants' demographics, seeking treatment, or its effect (p > 0.05).

**Table 2:** Relationship between DLQI levels and participants' demographic data, causes of alopecia, and seeking treatment and its effect (n = 231) DLQI: Dermatology Life Quality Index.

Variable	DLQI levels		χ2	p-value
	Small or no effect, No. (%)	Moderate effect or more, No. (%)		
Age	38.13 ± 15.34	37.93 ± 13.08	0.04	0.968
Gender			0.09	0.755
Female	85 (45)	20 (47.6)		
Male	104 (55)	22 (52.4)		
Marital status			2.15	0.541
Widow	1 (0.5)	0 (0.0)		
Single	64 (33.9)	12 (28.6)		
Married	121 (64)	28 (66.7)		
Divorced	3 (1.6)	2 (4.8)		
Employment			4.27	0.511
Housewife	23 (12.2)	6 (14.3)		
Student	47 (24.9)	8 (19)		
Unemployed	12 (6.3)	5 (11.9)		
Retired	37 (19.6)	7 (16.7)		
Government employee	30 (15.9)	10 (23.8)		
Private sector employee	40 (21.2)	6 (14.3)		
Monthly income (Saudi Riyal)			1.16	0.884
<2000	64 (33.9)	16 (38.1)		
2000-<5000	18 (9.5)	5 (11.9)		
5000-<10000	32 (16.9)	6 (14.3)		
10000-15000	22 (11.6)	3 (7.1)		
>15000	53 (28)	12 (28.6)		
Education			0.69	0.983
Primary	1 (0.5)	0 (0.0)		
Middle	6 (3.2)	1 (2.4)		
Secondary	53 (28)	10 (23.8)		
Diploma	22 (11.6)	5 (11.9)		
Bachelor	91 (48.1)	22 (52.4)		
Postgraduate	16 (8.5)	4 (9.5)		
Causes of alopecia			9.84	0.043
Malnutrition	29 (15.3)	15 (35.7)		
Hereditary baldness	101 (53.4)	18 (421.9)		
Telogen effluvium	14 (7.4)	3 (7.1)		
Psoriasis	2 (1.1)	0 (0.0)		
I don't know the type	43 (22.8)	6 (14.3)		
Have you searched for a treatment for this problem?			1.24	0.264
No	99 (52.4)	18 (42.9)		
Yes	90 (47.6)	24 (57.1)		
If yes, did you benefit from the treatment?			1.95	0.376
No	74 (39.2)	18 (42.9)		
Yes	16 (8.5)	6 (14.3)		

Table 3 shows that patients who had higher depression levels had a significantly younger mean age, being females, married, and students (p < 0.05). At the same time, depression levels were significantly higher among patients with a monthly income <2000 SR, who had hereditary baldness, and who searched for treatment but did not benefit from treatment (p < 0.05).

**Table 3:** Relationship between depression levels and participants' demographic data, causes of alopecia, and seeking treatment and its effect (n = 231) PHQ-9: Patient Health Questionnaire-9.

Variable	PHQ-9 levels		χ2	p-value
	Minimal depression, No. (%)	Higher depression, No. (%)		
Age	44.29 ± 15.13	32.41 ± 12.27	5.94	<0.001
Gender			17.92	<0.001
Female	34 (30.9)	71 (58.7)		
Male	67 (69.1)	50 (41.3)		
Marital status			15.51	0.001
Widow	0 (0.0)	1 (0.8)		
Single	26 (23.6)	50 (41.3)		
Married	84 (76.4)	65 (53.7)		
Divorced	0 (0.0)	5 (4.1)		
Employment			23.85	<0.001
Housewife	9 (8.2)	20 (16.5)		
Student	20 (18.2)	35 (28.9)		
Unemployed	5 (4.5)	12 (9.9)		
Retired	34 (30.9)	10 (8.3)		
Government employee	20 (18.2)	20 (16.5)		
Private sector employee	22 (20)	24 (19.8)		
Monthly income (Saudi Riyal)			22.93	<0.001
<2000	26 (23.6)	54 (44.6)		
2000-<5000	7 (6.4)	16 (13.2)		
5000-<10000	18 (16.4)	20 (16.5)		
10000-<15000	14 (12.7)	11 (9.1)		
>15000	45 (40.9)	20 (16.5)		
Education			9.06	0.107
Primary	1 (0.9)	0 (0.0)		
Middle	2 (1.8)	5 (4.1)		
Secondary	23 (20.9)	40 (33.1)		
Diploma	17 (15.5)	10 (8.3)		
Bachelor	55 (50)	58 (47.9)		
Postgraduate	12 (10.9)	8 (6.6)		
Causes of alopecia			14.64	0.006
Malnutrition	11 (10)	33 (27.3)		
Hereditary baldness	66 (60)	53 (43.8)		
Telogen effluvium	7 (6.4)	10 (8.3)		
I don't know the type	26 (23.6)	23 (19)		
Psoriasis	0 (0.0)	2 (1.7)		
Have you searched for a treatment for this problem?			7.34	0.007
No	66 (60)	51 (42.1)		
Yes	44 (40)	70 (57.9)		
If yes, did you benefit from the treatment?			7.84	0.02
No	37 (33.6)	55 (45.5)		
Yes	7 (6.4)	15 (12.4)		

Table 4 shows that a highly significant negative correlation was found between participants' age and their PHQ-9 scores (r = -0.52, p < 0.001) and their self-esteem scores (r = 0.34, p < 0.001).

**Table 4:** Spearman's correlation analysis between participants' age and their PHQ-9, DLQI, and self-esteem scores PHQ-9: Patient Health Questionnaire-9; DLQI: Dermatology Life Quality Index.

Variable	Age	
	r	p-value
PHQ-9 scores	-0.52	<0.001
DLQI scores	-0.09	0.161
Self-esteem	0.34	<0.001

**Figure 4:**
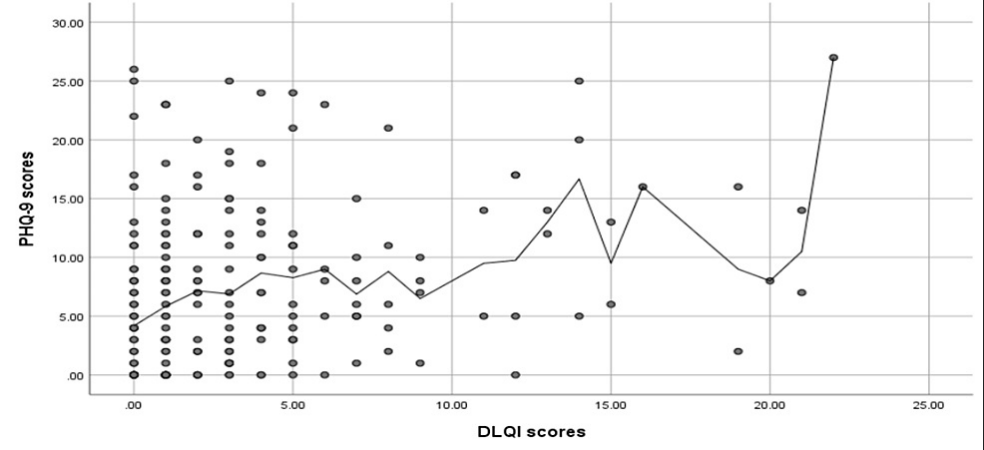
Spearman's correlation analysis between the PHQ-9 scores and the DLQI scores PHQ-9: Patient Health Questionnaire-9; DLQI: Dermatology Life Quality Index.

**Figure 5:**
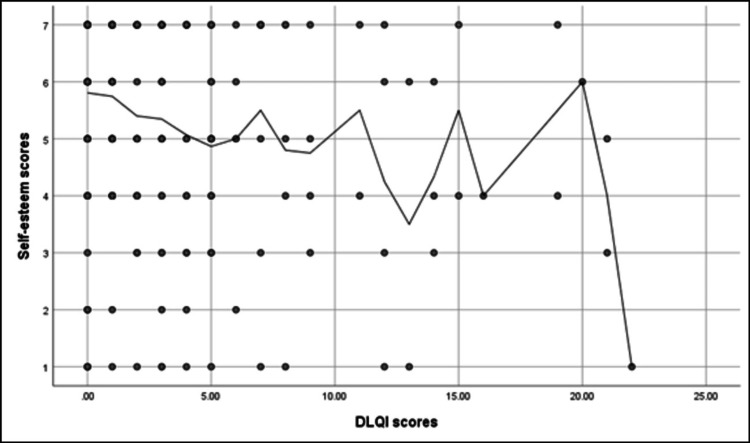
Spearman's correlation analysis between the DLQI scores and the self-esteem scores DLQI: Dermatology Life Quality Index.

**Figure 6:**
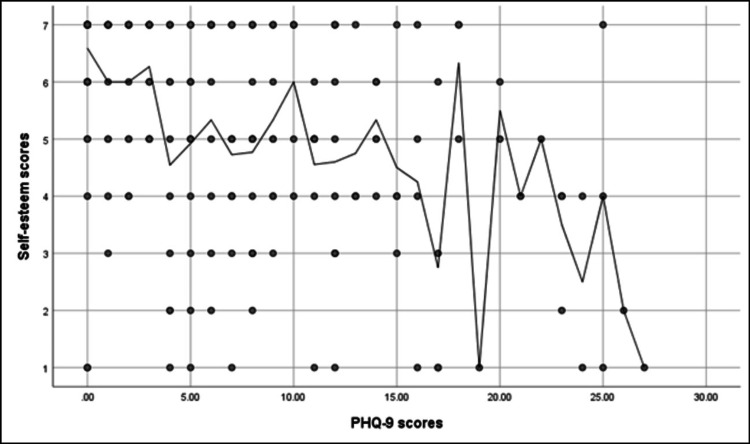
Spearman's correlation analysis between the PHQ-9 scores and the self-esteem scores PHQ-9: Patient Health Questionnaire-9.

Figure 4 shows that a significant positive correlation was found between the PHQ-9 scores and the DLQI scores (r = 0.36, p < 0.001). While a significant negative correlation was found between the DLQI score and self-esteem scores (r = -0.23, p < 0.001) (Figure 5). A significant negative correlation was found between the PHQ-9 scores and self-esteem scores (r = -0.52, p < 0.001) (Figure 6).

## Discussion

The present study aimed to investigate the prevalence of depression and its impact on the quality of life of patients with alopecia. Our results showed that over half of the participants had higher levels of depression (52.4%), while 47.6% had minimal depression. This finding is consistent with previous studies that reported a high prevalence of depression in patients with alopecia [13,14]. Furthermore, our study revealed that depression was significantly higher among female patients, married individuals, students, and those with a younger mean age. Patients with a monthly income <2000 SR, hereditary baldness, seeking treatment, and benefiting from it also had significantly higher levels of depression.

Regarding the causes of alopecia, hereditary baldness was the most common cause among our participants (51.5%), while 49.4% sought therapy, and only 9.5% benefited from it. This finding aligns with previous studies that reported a high prevalence of androgenetic alopecia among both genders [15]. Additionally, individuals who reported a moderate or greater impact on their lives because of alopecia were more likely to have hereditary baldness. This finding indicates that people with a genetic predisposition to alopecia are at a higher risk of developing depression and experiencing adverse effects on their quality of life. Therefore, it is essential to address the emotional and psychological impact of alopecia in people with hereditary baldness to enhance their overall well-being.

Concerning the quality of life, our study found that 18.2% of the participants had a notable impact on their lives due to alopecia, while 81.8% had little or no effect. Additionally, the percentage of patients who experienced a moderate or higher effect on their lives due to alopecia was significantly higher than those with hereditary baldness. Our results are similar to those found in the study conducted by Marahatta et al. in Nepal regarding the impact of alopecia on quality of life. In their study, they found that patients with alopecia areata frequently experience psychological problems, which may impact their quality of life. However, they did not specifically mention the impact of hereditary baldness on quality of life [13].

The average score for self-esteem among the participants was 5.41, and the standard deviation was 1.82. A majority of the participants, comprising 44.2% of the total, rated their self-esteem as 7.

Additionally, our study found significant correlations between the participants' age, self-esteem scores, PHQ-9 scores, and DLQI scores. The negative correlation between age and PHQ-9 scores and self-esteem scores suggests that older participants experienced less depression and higher self-esteem. In contrast, the positive correlation between PHQ-9 scores and DLQI scores suggests that depression may negatively affect the quality of life. Furthermore, the negative correlation between DLQI scores and self-esteem scores suggests that participants with lower quality of life may have lower self-esteem, and the negative correlation between PHQ-9 scores and self-esteem scores indicates that depression may lower self-esteem. Overall, the study suggests that there may be complex relationships between depression, quality of life, self-esteem, and age. This finding is consistent with a previous study conducted by Karia et al. in India that points out that quality of life scores were significantly correlated with the existence of psychiatric conditions [15].

Study strengths and limitations

One of the main strengths of this study is its use of standardized and validated instruments for assessing the health-related quality of life, depression, and self-esteem in participants with alopecia. Study design, which is a cross-sectional study, allows for the collection of data from a large sample size in a relatively short period of time. Additionally, the study utilized an online-based questionnaire, which provided a convenient and accessible method of data collection. Furthermore, the study was reviewed and approved by the Institutional Review Board Committee at Imam Abdulrahman Bin Faisal University, ensuring ethical considerations were met.

One limitation of the study is the voluntary response sampling technique used, which may lead to selection bias, as only individuals who are willing to participate will respond to the questionnaire. Additionally, the study was conducted in a specific geographical location, which limits the generalizability of the findings to other populations. Another limitation is the reliance on self-reported data, which may lead to social desirability bias and recall bias. Finally, due to the cross-sectional design of the study, causality cannot be inferred, and the temporal relationship between alopecia, depression, self-esteem, and health-related quality of life cannot be established.

## Conclusions

In conclusion, our study highlights the high prevalence of depression among patients with alopecia and its significant impact on their quality of life. Patients with hereditary baldness are particularly vulnerable to depression and a negative impact on their quality of life. Hence, providing psychological support and counseling for these patients is crucial to improving their mental health and quality of life.

## Appendices

Questionnaire

1. Language preferred for questionnaire answering:

Arabic

English

2. Do you consent to participate in this research?

Yes 

No

Demographic data

In this section, you will be asked about your general information.

3. Age

4. Sex

Male

Female

5. Nationality

Saudi

Non-Saudi

6. Marital status

Married

Single

Divorced

Widow 7. Occupation

Governmental employee

Private sector employee

Unemployed

Student

Housewife

Retired

8. Monthly income

<2000 SR

2000 - 5000 SR

5000 - 10000 SR

10000 - 15000 SR

>15000 SR

9. Education

Illiterate

Primary school

Elementary school

High school

Diploma

Bachelor

Postgraduate

In this section, you will be asked about the details of your condition.

10. Do you suffer from alopecia?

Yes

No

11. Which type of alopecia you have been diagnosed with?

If it is not listed below, choose "other" and type it.

Androgenic alopecia

Alopecia areata

Telogen effluvium

Traction alopecia

I don't suffer from alopecia

I don't know the type

Other:

12. Did you seek any medical treatment?

Yes

No

13. If you answered yes to the previous question, did you benefit from the treatment?

Yes

No

Dermatology Life Quality Index [10].

The aim of this questionnaire is to measure how much your skin problem has affected your life OVER THE LAST WEEK. Please check one box for each question.

14. Over the last week, how itchy, sore, painful, or stinging has your skin been?

Very much

A lot

A little

Not at all

15. Over the last week, how embarrassed or self-conscious have you been because of your skin?

Very much

A lot

A little

Not at all

16. Over the last week, how much has your skin interfered with you going shopping or looking after your home or yard?

Very much

A lot

A little

Not at all

Not relevant

17. Over the last week, how much has your skin influenced the clothes you wear?

Very much

A lot

A little

Not at all

Not relevant

18. Over the last week, how much has your skin affected any social or leisure activities?

Very much

A lot

A little

Not at all

Not relevant

19. Over the last week, how much has your skin made it difficult for you to do any sport?

Very much

A lot

A little

Not at all

Not relevant

20. Over the last week, has your skin prevented you from working or studying?

Yes

No

Not relevant

21. If you answered the last question with "no," over the last week how much has your skin been a problem at work or studying?

A lot

A little

Not at all

22. Over the last week, how much has your skin created problems with your partner or any of your close friends or relatives?

Very much

A lot

A little

Not at all

Not relevant

23. Over the last week, how much has your skin caused any sexual difficulties?

Very much

A lot

A little

Not at all

Not relevant

24. Over the last week, how much of a problem has the treatment for your skin been, for example by making your home messy or by taking up time?

Very much

A lot

A little

Not at all

Not relevant

Patient Health Questionnaire-9 (PHQ-9) [11,16]

Over the last two weeks, how often have you been bothered by the following problems?

25. Little interest or pleasure in doing things

Not at all

Several days

More than half the days

Nearly every day

26. Feeling down, depressed, or hopeless

Not at all

Several days

More than half the days

Nearly every day

27. Trouble falling or staying asleep, or sleeping too much

Not at all

Several days

More than half the days

Nearly every day

28. Feeling tired or having little energy

Not at all

Several days

More than half the days

Nearly every day

29. Poor appetite or overeating

Not at all

Several days

More than half the days

Nearly every day

30. Feeling bad about yourself - or that you are a failure or have let yourself or your family down

Not at all

Several days

More than half the days

Nearly every day

31. Trouble concentrating on things, such as reading the newspaper or watching television

Not at all

Several days

More than half the days

Nearly every day

32. Moving or speaking so slowly that other people could have noticed, or so fidgety or restless that you have been moving a lot more than usual

Not at all

Several days

More than half the days

Nearly every day

33. Thoughts that you would be better off dead, or thoughts of hurting yourself in some way

Not at all

Several days

More than half the days

Nearly every day

Single Item Self-esteem Scale [12]

34. I have high self-esteem

Not very true of me

1

2

3

4

5

6

7

Very true of me
